# Pex11a deficiency causes dyslipidaemia and obesity in mice

**DOI:** 10.1111/jcmm.14108

**Published:** 2018-12-25

**Authors:** Congcong Chen, Hongwei Wang, Bicheng Chen, Deyuan Chen, Chaosheng Lu, Haiyan Li, Yan Qian, Yi Tan, Huachun Weng, Lu Cai

**Affiliations:** ^1^ Chinese‐American Research Institute for Pediatrics & Department of Pediatrics The First Affiliated Hospital of Wenzhou Medical University Chashan University‐Town Wenzhou China; ^2^ Hepatobiliary and Pancreatic Surgery Laboratory The First Affiliated Hospital of Wenzhou Medical University Wenzhou China; ^3^ Department of Pathology The First Affiliated Hospital of Wenzhou Medical University Wenzhou China; ^4^ Department of Pharmaceutical Sciences Wenzhou Medical University Wenzhou China; ^5^ Department of Pharmacy Jinhua Central Hospital Jinhua China; ^6^ Pediatric Research Institute Departments of Pediatrics Radiation Oncology, Pharmacology and Toxicology University of Louisville Louisville Kentucky

**Keywords:** adipose tissue, fatty acid metabolism, lipogenesis, obesity, peroxisomes

## Abstract

Peroxisomes play a central role in lipid metabolism. We previously demonstrated that Pex11a deficiency impairs peroxisome abundance and fatty acid β‐oxidation and results in hepatic triglyceride accumulation. The role of Pex11a in dyslipidaemia and obesity is investigated here with Pex11a knockout mice (Pex11a^−/−^). Metabolic phenotypes including tissue weight, glucose tolerance, insulin sensitivity, cholesterol levels, fatty acid profile, oxygen consumption, physical activity were assessed in wild‐type (WT) and Pex11a^−/−^ fed with a high‐fat diet. Molecular changes and peroxisome abundance in adipose tissue were evaluated through qRT‐PCR, Western blotting, and Immunofluorescence. Pex11a^−/−^ showed increased fat mass, decreased skeletal muscle, higher cholesterol levels, and more severely impaired glucose and insulin tolerance. Pex11a^−/−^ consumed less oxygen, indicating a decrease in fatty acid oxidation, which is consistent with the accumulation of very long‐ and long‐chain fatty acids. Adipose palmitic acid (C16:0) levels were elevated in Pex11a^−/−^, which may be because of dramatically increased fatty acid synthase mRNA and protein levels. Furthermore, Pex11a deficiency increased ventricle size and macrophage infiltration, which are related to the reduced physical activity. These data demonstrate that Pex11a deficiency impairs physical activity and energy expenditure, decreases fatty acid β‐oxidation, increases de novo lipogenesis and results in dyslipidaemia and obesity.

## 1 INTRODUCTION

Obesity is a significant risk factor for many life‐threatening diseases, such as type II diabetes, hypertension, cardiovascular and cerebrovascular disease, and its global impact on health is enormous.^1^ Obesity can begin as a simple overstorage of unmetabolized energy in the adipose tissue of individuals whose caloric intake exceeds the energy combustion capability of peroxisome proliferator‐activated receptor‐(PPAR)‐mediated fatty acid oxidation systems.^2^ Furthermore, adipogenesis is driven by the activation of a cascade of genes, especially PPARγ.^3^, ^4^

Peroxisomes are single‐membrane‐bound organelles present in all mammalian cells, initially identified in kidney and liver cells.^5^, ^6^ They participate in lipid metabolism and are indispensable for the degradation of carboxylates via alpha and beta‐oxidation, formation of conjugated bile acids from cholesterol and synthesis of certain polyunsaturated fatty acids and ether lipids, as well as H_2_O_2_ metabolism.^7^, ^8^, ^9^ Peroxisomes are highly versatile and dynamic organelles whose size, shape, number, and protein content vary according to the cell type, metabolic requirements and extracellular stimuli.^7^, ^10^, ^11^

Currently, two types of peroxisome biogenesis have been considered. Some studies propose that peroxisomes can form de novo from the endoplasmic reticulum via a maturation process.^12^, ^13^ Other studies have demonstrated that peroxisomes can also multiply through growth and division.^14^, ^15^, ^16^ Peroxisome proliferation can be divided into at least three distinct steps in mammalian cells, including elongation, segregation and constriction of the peroxisomal membrane, and division. The Pex11a gene is one of Pex11 gene family members involved in peroxisome elongation.^14^, ^16^, ^17^ We previously demonstrated that Pex11a knock out (Pex11a^−/−^) mice exhibit decreased peroxisome abundance and fatty acid β‐oxidation in the liver, accelerated bodyweight gain and hepatic triglyceride accumulation.^17^ However, whether Pex11a deficiency affects peroxisome abundance and lipid metabolism in adipose tissue is unclear.

In adipocytes, peroxisomes tend to be small in size and localized in the vicinity of lipid droplets. A large increase in peroxisome number was observed during the differentiation of 3T3‐L1 adipocytes.^18^ Several studies indicate that peroxisome metabolism is critical for normal adipose function.^19^, ^20^ Pex7‐deficient mice exhibited impaired peroxisome biogenesis, lacked plasmalogens, and had severely reduced adiposity.^20^ However, a recent study showed that knockdown of Pex16 reduced peroxisome number and peroxisomal fatty acid oxidation, thereby causing accumulation of very long‐ and long‐chain fatty acids in 3T3‐L1 cells.^21^ Furthermore, mice specifically lacking Pex5 in adipose tissue showed an increase in fat mass as a result of reduced lipolysis.^19^ The expression of Pex genes involved in peroxisomal biogenesis is significantly increased during differentiation of brown adipocytes in culture and in brown adipose tissue (BAT) in mice exposed to cold temperatures in a manner dependent on PPARγ coactivator‐1α.^22^ These studies demonstrate that peroxisome biogenesis is important in lipid metabolism of adipose tissue. In the present study, we used Pex11a^−/−^ mice to investigate the role of Pex11a in lipid metabolism and obesity. Understanding of the molecular and physiological functions of Pex11 gene and etiology of dyslipidaemia and obesity, will facilitate the development of therapeutic strategies of targeting Pex11a for dyslipidaemia and obesity.

## 2 MATERIAL AND METHODS

### 2.1 Animal studies and diets

Pex11a^−/−^ mice were generated as described previously 17 and gifted from Laboratory Animal Resource Bank of the National Institutes of Biomedical Innovation, Health and Nutrition (Osaka, Japan). Heterozygous mice were intercrossed to produce homozygous Pex11a^−/−^ mice. These homozygous Pex11a^−/−^ mice were backcrossed with C57BL mice [wild‐type (WT) mice purchased from the Experimental Animal Center of Beijing University of Medical Science (Beijing, China) and allowed to acclimate for 2 weeks] five times prior to the generation of Pex11a^−/−^ mice. Their offspring (Pex11a^−/−^ and WT mice) were bred in the same temperature‐controlled pathogen‐free room with lights on from 07:00 to 19:00 (daytime) and were used in the present experiment. The experimental protocols were approved by the Wenzhou Medical University Committee for Laboratory Animals, and all animal treatment protocols were consistent with the National Institutes of Health Guide for the Care and Use of Laboratory Animals. Mice were allowed ad libitum access to food and water. The effects of a chow diet consisting of 24.9% protein, 4.6% fat (calorie ratio 12%) and 51.4% carbohydrate (wt/wt) were compared with those of a high‐fat diet (HFD) consisting of 23% protein, 35% fat (calorie ratio 62.2%) and 25.3% carbohydrate. Male mice fed a chow diet were scarified at the age of 20 weeks; male mice fed an HFD for 8 weeks were scarified at the age of 14 weeks.

Whole‐body energy metabolism was evaluated using a Lab Animal Monitoring System (CLAMS, Columbus Instruments, Columbus, OH, USA). Mice were acclimated to the metabolic chambers for 2 days before the beginning the experiment to minimize stress from the housing change. O_2_ consumption, food intake and movement indices were collected every 15 minutes for each mouse.

To assess glucose tolerance, mice were fasted overnight (16 hours). After their fasting glucose levels were measured, mice were injected intraperitoneally with glucose (2.5 g/kg bodyweight). Next, blood glucose levels were measured at 15, 30, 60, 90, 120 and 180 minutes after injection. Blood glucose was measured by using a blood glucose monitoring system (Nipro, Osaka, Japan) according to the manufacturer's instructions. For the insulin tolerance test, 6‐hour fasted mice were injected intraperitoneally with insulin (0.75 mU/g bodyweight), and blood glucose levels were measured as described above.

The ventricles of the 20‐week‐old male WT mice and Pex11a^−/−^ mice fed a chow diet were determined by 7T magnetic resonance imaging and then scarified (MRI; Bruker, Germany). Motor function was examined using the classic rotarod test. The accelerated rotarod is electronically controlled and started by manual switches (Ugo Basile, Varese, Italy). It consists of five plastic rods with a knurled surface for the mice to grip and flanges on both sides to confine the mouse on its own rod. Mice were placed on the rod in such a way that they were allowed to walk forward. Mice were trained to adapt to the apparatus at the speed of 25 rpm for 3 days before formal testing. After starting the apparatus, the time at which the individual mouse fell off were recorded.

Under fasting conditions, WT mice and Pex11a^−/−^ mice were deprived of food but provided water ad libitum for 24 hours starting at the beginning of the light cycle (7 am). At the termination of the experiments, mice were subsequently euthanized under sodium pentobarbital anaesthesia. Blood was collected from the abdominal vein and centrifuged for 10 minutes to collect serum. Serum was kept at −80°C. BAT, epididymal white adipose tissue (eWAT) and liver were removed and kept at −80°C.

### 2.2 Metabolite assays

Serum total cholesterol, very low‐density lipoprotein cholesterol (VLDL‐C), low‐density lipoprotein cholesterol (LDL‐C), high‐density lipoprotein cholesterol (HDL‐C) and insulin levels were measured with a Cholesterol Quantitation Kit, a VLDL‐C Quantitation Kit, an LDL‐C Quantitation Kit, an HDL‐C Quantitation Kit and an insulin ELISA Kit (Nanjing Jiancheng Bioengineering Institute; Nanjing, China), respectively. For all of the assays, the samples were run in duplicate, and the results were averaged.

For the fatty acid profile, 40 ± 1 mg of frozen liver or eWAT was homogenized in 500 μL of methanol in an ice bath by using a TissueLyser (JX‐24, Jingxin, Shanghai, China) with zirconia beads for 3 minutes at 30 Hz. After centrifugation at 14 000× *g* and 4°C for 15 minutes, supernatants were collected. Volumes of 80 μL of liver supernatant, 50 μL of eWAT supernatant or 10 μL of serum were combined with 10 μL of 250 μg/mL nonadecanoic acid, and the mixtures were methylated in PTFE screw‐capped glass vials with 1 mL of 10% methanolic acetyl chloride and 250 μL of n‐hexane at room temperature overnight. Then, 5 mL of 6% potassium carbonate solution was added to each vial. The hexane extraction (150 μL) was collected, and anhydrous sodium sulphate (20 mg) was used to remove traces of water prior to performing GC‐MS analysis. A reference material, 37 Component FAME Mix standard solution, was diluted from 20 μg/mL to 0.001–10 μg/mL. Then, 40 μL of the diluted standard solution and 40 μL of 20 μg/mL nonadecanoic acid were combined prior to performing GC‐MS analysis. Instrumental analysis was performed on an Agilent 7890A gas chromatography system coupled to an Agilent 5975C inert MSD system (Agilent Technologies Inc., Palo Alto, CA, USA). A TRACE TR‐FAME fused‐silica capillary column (10 m × 0.1 mm × 0.2 μm; Thermo Fisher Scientific, Waltham, MA, USA) was utilized to separate the derivatives. Helium (>99.999%) was used as a carrier gas at a constant flow rate of 0.35 mL/min through the column. The solvent delay time was 1.5 minutes. The initial oven temperature was held at 40°C for 1 minute, ramped to 150°C at a rate of 80°C/min, ramped to 240°C at a rate of 8°C/min and finally held at 240°C for 1 minute. The temperatures of the injector, transfer line, and electron impact ion source were set to 220°C, 250°C and 230°C, respectively. The impact energy was 70 eV, and data were collected in a SIM mode (m/z 50‐550).

### 2.3 Real‐time PCR analysis

Total RNA was isolated using the TRIzol method (Invitrogen, Carlsbad, CA, USA) according to the manufacturer's instructions. For real‐time PCR analysis, cDNA obtained by reverse transcription was amplified using the appropriate primers, and mRNA expression levels were determined by real‐time RT‐PCR using a commercial kit (Applied Biosystems, Foster City, CA, USA). The primer sequences used are shown in Table S1. All samples were analysed in triplicate multiplex reactions, which measured both the gene of interest and glyceraldehyde 3‐phosphate dehydrogenase (GAPDH) as an internal control.

### 2.4 Western blotting

For peroxisome membrane protein 70 (PMP70) and fatty acid synthase **(**FAS) immunoblotting, 100 mg of BAT was homogenized in RIPA buffer (0.5% NP‐40, 0.1% sodium deoxycholate, 150 mmol L^−1^ NaCl, 50 mmol L^−1^ Tris‐Cl, pH 7.5). After centrifugation at 15 000× *g* for 10 minutes at 4°C, the supernatant was collected and kept at −80°C. Lysates were resolved by SDS‐PAGE, transferred to PVDF membrane (Milipore, Billerica, MA, USA), and probed with anti‐PMP70 (Abcam, Cambridge, UK), anti‐FAS (Cell Signaling Technology, Danvers, MA, USA) and anti‐β‐actin (Santa Cruz Biotechnology, Santa Cruz, CA, USA).

### 2.5 Histological analysis

Immunofluorescence and peroxisome abundance detection were performed as described previously.^17^ BAT was fixed in 4% paraformaldehyde overnight and rinsed with phosphate‐buffered saline. The sections were incubated with rabbit anti‐PMP70 (Abcam) primary antibodies overnight at 4°C, followed by Alexa Fluor 488‐conjugated goat anti‐rabbit secondary antibodies (Abcam) for 60 minutes at room temperature. Fluorescence images were acquired using a confocal laser‐scanning microscope (Leica, Wetzlar, Germany).

Immunohistochemical staining was performed as described previously.^26^ Deparaffinized brain sections were heated for 20 minutes at 121°C in 10 mmol L^−1^ citric acid solution for antigen retrieval and then incubated with antibody against F4/80 (Santa Cruz Biotechnology). The primary antibody was detected using the a Histofine Simple Stain MAX‐PO (mouse) kit (Nichirei, Tokyo, Japan) and peroxidase stain DAB kit (Nacalai Tesque, Kyoto, Japan).

### 2.6 Statistical analysis

Results are expressed as the mean ± SD. The data were analysed using one‐way analysis of variance (ANOVA) and a 2‐tailed Student's *t*‐test. P values <0.05 were considered indicative of statistically significant differences.

## 3 RESULTS

### 3.1 Pex11a deficiency results in adiposity, dyslipidaemia and loss of glycemic control

**Figure 1:**
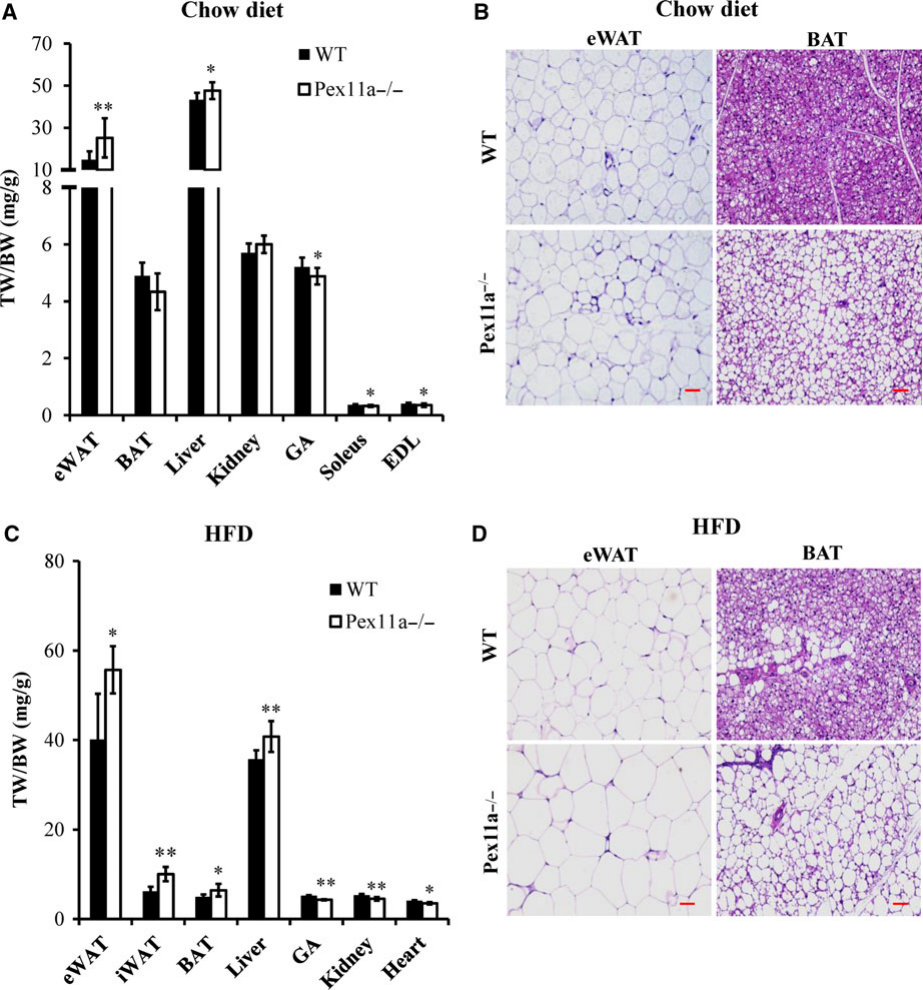
Pex11a deficiency increases adiposity. Tissue weights of WT and Pex11a^−/−^ mice fed with (A) a chow diet or (C) a high‐fat diet (HFD) for 8 weeks (Values are mean ± SD, n = 6 per group, *P < 0.05, **P < 0.01 vs WT). H&E staining of epididymal white adipose tissue (eWAT) and brown adipose tissue (BAT) of WT and Pex11a^−/−^ mice fed with a chow diet (B) or an HFD for 8 weeks (D). Scale bar, 100 μM; TW, tissue weight (mg); BW, bodyweight (g); iWAT, inguinal white adipose tissue; GA, gastrocnemius; EDL, extensor digitorum longus

Pex11a has been reported to have a tissue specific expression,^23^, ^24^ and the relative Pex11a mRNA levels are expressed prominently in the adipose tissue, liver, kidney, heart, gastrocnemius and brain (data not shown). Our previous studies showed that Pex11a play an important role in the etiological process of liver and kidney and the bodyweights of Pex11a^−/−^ mice were significantly higher than those of WT mice irrespective of a chow diet or HFD.^17^, ^25^ In present study, increased ratios of eWAT and liver weights to bodyweight (Figure 1A and C) but decreased ratios of muscle mass (gastrocnemius, soleus, tibialis anterior) to bodyweight in Pex11a^−/−^ mice were observed. On an HFD, there were increased ratio of BAT (scapular) and inguinal WAT to bodyweight but decreased ratios of kidney and heart weights to bodyweight in Pex11a^−/−^ mice (Figure 1C). These results indicate that increased bodyweights of Pex11a^−/−^ mice may be because of increased adipose tissue mass. Histological analysis of eWAT and BAT indicated that the expansion of eWAT and BAT was associated with an enlargement of adipocytes (Figure 1B and D).

**Figure 2:**
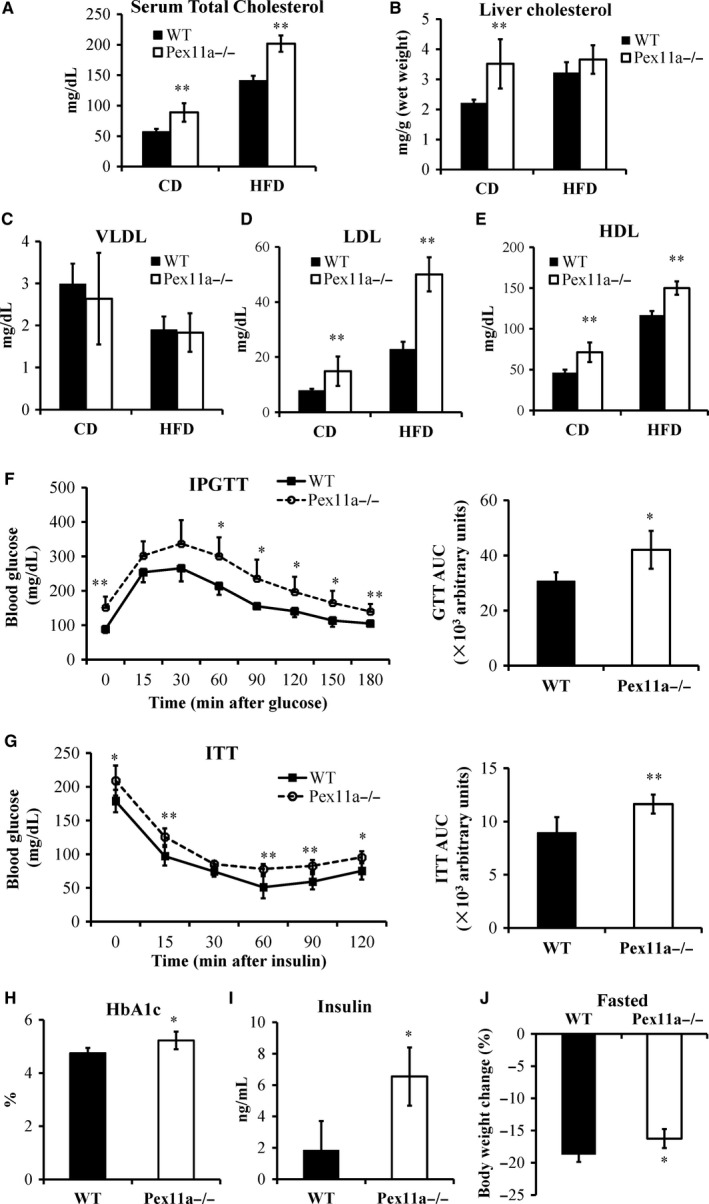
Pex11a deficiency results in dyslipidaemia and loss of glycemic control. Serum (A) and hepatic (B) cholesterol levels were measured in WT and Pex11a^−/−^ mice fed with a chow diet (CD) or a high‐fat diet (HFD) for 8 weeks. Very low‐density lipoprotein (VLDL, C), low‐density lipoprotein (LDL, D) and high‐density lipoprotein (HDL, E) levels in sera were measured. n = 8 per group. (F) Glucose tolerance test (IPGTT) and area under curve (AUC) of GTT. Blood glucose levels were measured in 8‐week high‐fat diet‐fed WT and Pex11a^−/−^ mice after an overnight fast and at the indicated times after an intraperitoneal injection of glucose. n = 8 per group. (G) Insulin tolerance test (ITT) and AUC of ITT. Blood glucose levels were measured after a 6‐h fast and at the indicated times after an intraperitoneal injection of insulin in mice fed with an HFD for 8 weeks. n = 8 per group. Serum haemoglobin A1c (HbA1c; H) and insulin (I) levels were measured in mice fed with an HFD for 8 weeks. n = 8 per group. (J) Bodyweight change (%). WT and Pex11a^−/−^ mice fed with a chow diet were fasted for 24‐h. % change from baseline is shown. n = 8 per group. Values are mean ± SD, *P < 0.05, **P < 0.01 vs WT

Although there were no differences in serum triglyceride between the genotypes at our previous study,^17^ serum total cholesterol, LDL, and HDL levels in Pex11a^−/−^ mice were significantly elevated on a chow diet or HFD (Figure 2). The hepatic total cholesterol levels in Pex11a^−/−^ mice were significantly higher than those in WT mice on a chow diet but not on an HFD (Figure 2B).

As in our previous studies, on a chow diet, there were no differences in blood glucose levels and glucose tolerance between WT and Pex11a^−/−^ mice.^17^ However, feeding an HFD elicited a phenotypic difference. The levels of fasting glucose and haemoglobin A1c in Pex11a^−/−^ mice were higher than those in WT mice (Figure 2F and H). Pex11a^−/−^ mice had significantly worse glucose tolerance compared with WT mice, which is accompanied with higher serum insulin levels (Figure 2F and I). The insulin tolerance test showed that Pex11a^−/−^ mice have lower sensitivity to insulin than WT mice (Figure 2G).

### 3.2 Pex11a deficiency alters the fatty acid profile

**Figure 3:**
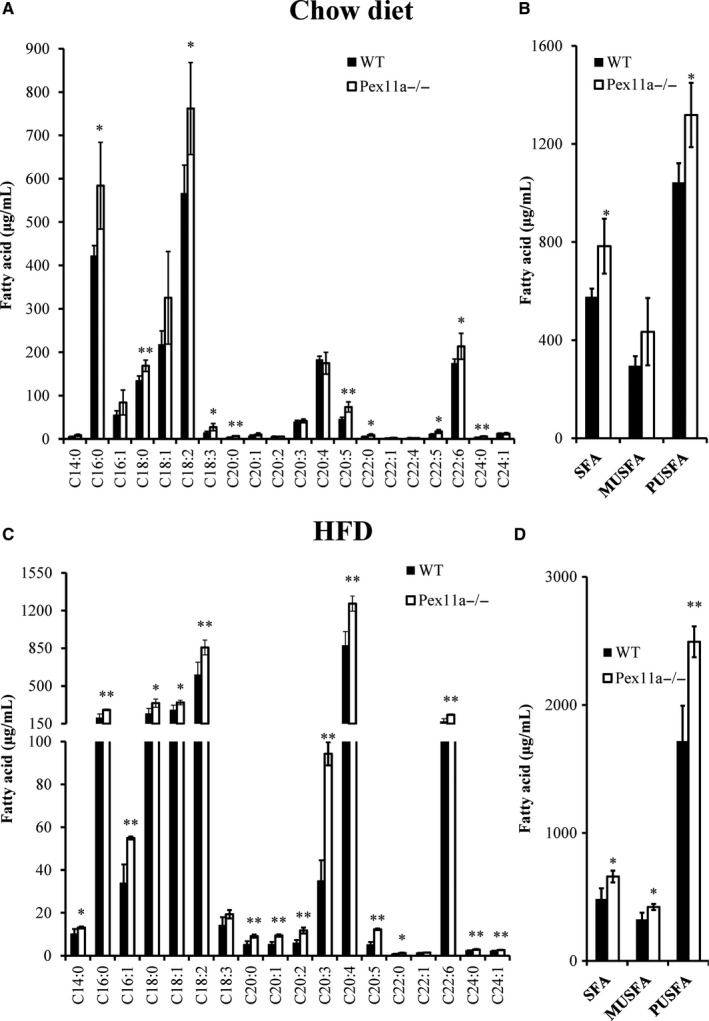
Pex11a deficiency alters the serum fatty acid profile. Serum fatty acid profiles in WT and Pex11a^−/−^ mice fed with a chow diet (A and B) or a high fat diet (HFD; C and D) for 8 weeks were determined by GC‐MS. Relative signal intensities were normalized to an internal standard (C19:0). B and D: Sum concentration of fatty acids by degree of saturation. SFA, saturated fatty acids; MUSFA, monounsaturated fatty acids; PUSFA, polyunsaturated fatty acids. Values are mean ± SD, n = 4 per group, *P < 0.05, **P < 0.01 vs WT

Stored energy in adipose tissue is released and oxidized for survival during starvation. The mice were fasted for 24 hours. The results showed that the change in bodyweight in WT mice was greater than that in Pex11a^−/−^ mice (Figure 2J), indicating that fatty acid oxidation may be impaired in Pex11a^−/−^ mice. Peroxisomes play an important role in β‐oxidation of fatty acids, especially very long‐chain fatty acids. To evaluate the effect of Pex11a deficiency on fatty acid β‐oxidation, fatty acid profiling was performed. GC‐MS analysis showed that serum levels of very long‐ and long‐chain saturated fatty acids (C16:0‐C24:0) in Pex11a^−/−^ mice were significantly higher than those in WT mice on a chow diet or HFD, especially levels of palmitic acid (C16:0) and stearic acid (C18:0) (Figure 3A and C). Monounsaturated fatty acid levels in Pex11a^−/−^ mice were significantly higher than in WT mice on an HFD but not a chow diet (Figure 3B and D). Whether under chow diet or HFD conditions, polyunsaturated fatty acid levels in Pex11a^−/−^ mice were higher than those in WT mice. An HFD lowered palmitic acid levels (WT 421.98 ± 23.48 μg/mL vs Pex11a^−/−^ 584.00 ± 100.18 μg/mL to 207.60 ± 33.35 μg/mL vs 279.26 ± 8.07 μg/mL) but increased cis‐5,8,11,14‐eicosatetraenoic acid levels (C20:4).

**Figure 4:**
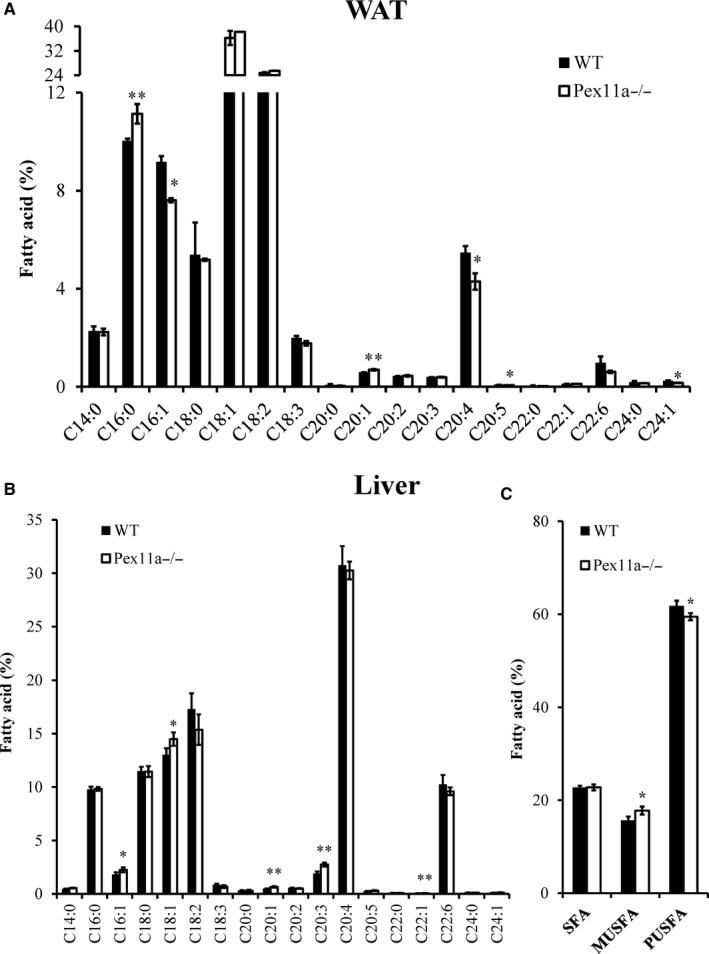
Pex11a deficiency alters fatty acid profiles of white adipose tissue (WAT) and liver. Fatty acid profiles of WAT (A) and liver (B) of WT and Pex11a^−/−^ mice fed with a high‐fat diet for 8 weeks were determined by GC‐MS. Relative signal intensities were normalized to an internal standard (C19:0) and expressed as percentage of total fatty acids. (C) Sum percentage of fatty acids by degree of saturation in the liver. SFA, saturated fatty acids; MUSFA, monounsaturated fatty acids; PUSFA, polyunsaturated fatty acids. Values are mean ± SD, n = 4 per group, *P < 0.05, **P < 0.01 vs WT

We also measured the fatty acid profile in eWAT. On an HFD, palmitic acid levels in Pex11a^−/−^ mice were significantly higher than those in WT mice (Figure 4A). However, cis‐5,8,11,14‐ eicosatetraenoic acid and palmitoleic acid (C16:1) levels in Pex11a^−/−^ mice were significantly lower than those in WT mice. The fatty acid profile in the liver showed that there were no differences in palmitic acid levels between WT and Pex11a^−/−^ mice. However, monounsaturated fatty acid levels in Pex11a^−/−^ mice were significantly higher than those in WT mice, especially palmitoleic acid (C16:1) and oleic acid (C18:1) (Figure 4B and C). However, hepatic polyunsaturated fatty acid levels in Pex11a^−/−^ mice were lower than those in WT mice.

These results indicate that Pex11a deficiency results in accumulation of very long‐ and long‐chain fatty acids in serum.

### 3.3 Pex11a deficiency impairs energy expenditure

**Figure 5:**
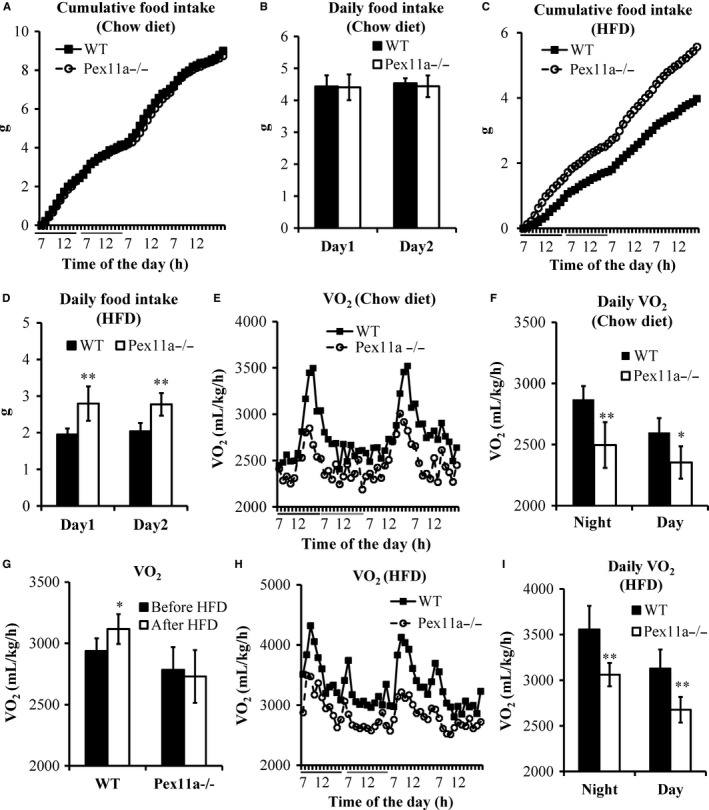
Pex11a deficiency increases food intake and decreases O_2_ consumption. Food intake was measured for 48 h in individually housed WT and Pex11a^−/−^ mice fed with a chow diet (A: Cumulative 48 h; the black lines and the grey lines indicate night and day, respectively. B: Daily food intake) or a high‐fat diet (HFD) for 8 weeks (C: Cumulative 48 h; the black lines and the grey lines indicate night and day, respectively. D: Daily food intake). O_2_ consumption (VO_2_) was measured for 48 h in mice fed with a chow diet (E: Continuous 48 h; the black lines and the grey lines indicate night and day, respectively. F: Night/day phase), a shift from chow diet to HFD (G: O_2_ consumption indices were collected in mice fed with a chow diet for 24 h and then fed an HFD for 24 h), and an HFD for 8 weeks (H: Continuous 48 h; the black lines and the grey lines indicate night and day, respectively. I: Night/day phase) using a Lab Animal Monitoring System. O_2_ consumption is reported as mL/kg (bodyweight)/h. Values are mean ± SD, n = 5 per group, *P < 0.05, **P < 0.01 vs WT

To evaluate the adiposity of Pex11a^−/−^ mice, we measured food intake and energy expenditure. On a chow diet, there were no differences in cumulative ad libitum food intake between the genotypes. However, on an HFD, increased food intake was observed in Pex11a^−/−^ mice (Figure 5C and D).

As mentioned above, Pex11a deficiency impairs fatty acid β‐oxidation, and the process of fatty acid β‐oxidation needs oxygen consumption; therefore, the effect of Pex11a deficiency on oxygen consumption in mice was evaluated. On a chow diet, Pex11a^−/−^ mice had decreased oxygen consumption compared to WT mice, especially during the night (Figure 5E and F). At the initial stage of HFD load, oxygen consumption was significantly increased in WT mice but not in Pex11a^−/−^ mice (Figure 5G). After 8 weeks of HFD, whether during the night or day, the levels of oxygen consumption in Pex11a^−/−^ mice were significantly lower than those in WT mice (Figure 5H and I). These results indicate that the Pex11a deficiency impairs oxygen consumption, may be because of decreased fatty acid β‐oxidation.

### 3.4 Pex11a deficiency impairs physical activity

**Figure 6:**
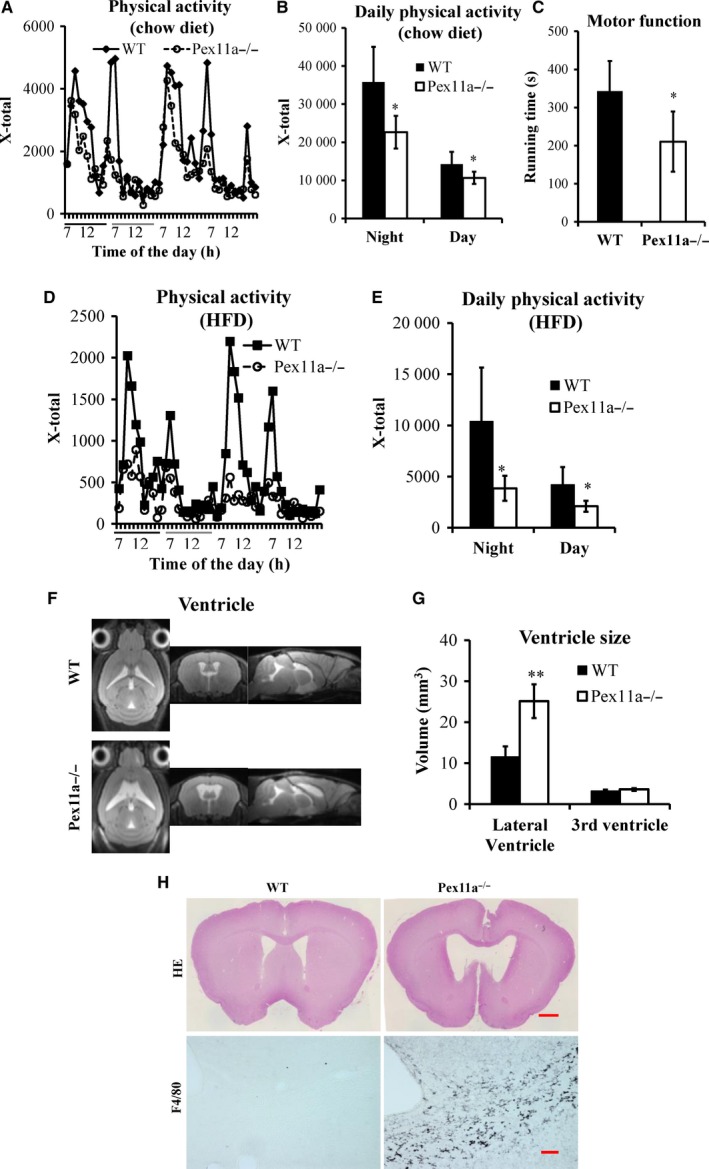
Pex11a deficiency impairs physical activity and increased lateral ventricle size and inflammation. Physical activity was measured using a Lab Animal Monitoring System in WT and Pex11a^−/−^ mice fed with a chow diet (A: Continuous 48 h; the black lines and the grey lines indicate night and day, respectively. B: Night/day phase) or a high‐fat diet (HFD) for 8 weeks (D: Continuous 48 h; the black lines and the grey lines indicate night and day, respectively. E: Night/day phase), n = 5 per group. (C) Motor function of mice fed with a chow diet was measured by the rotarod test, n = 5 per group. (F) Pictures of ventricles in WT and Pex11a^−/−^ mice fed with a chow diet were captured by MRI. (G) The volumes of lateral and third ventricles in F were analysed. n = 5 per group. (H) Cerebral sections from WT and Pex11a^−/−^ mice fed with a chow diet were stained with H&E (Scale bar: 500 μM) and for macrophages with antibodies against F4/80 in corpus callosum (Scale bar: 200 μM). Values are mean ± SD, *P < 0.05, **P < 0.01 vs WT

Whether during the night or day, on a chow diet, physical activity in Pex11a^−/−^ mice was significantly lower than that in WT mice, especially during the shift from night to day (Figure 6A and B). Furthermore, their motor function was examined using the classic rotarod test. The average rotarod running time of Pex11a^−/−^ mice was significantly shorter than that of WT mice (Figure 6C). HFD decreased physical activity in both the genotypes, especially in Pex11a^−/−^ mice. On an HFD, Pex11a^−/−^ mice had lower physical activity than WT mice, especially during the night (Figure 6D and E). These results indicate that Pex11a deficiency impairs physical activity, which is consistent with the decreased muscle mass (gastrocnemius, soleus and tibialis anterior) in Pex11a^−/−^ mice.

### 3.5 Pex11a deficiency results in larger lateral ventricle and increased macrophage infiltration

The observation of decreased physical activity and motor coordination prompted us to test the hypothesis that Pex11a deficiency may affect the formation and/or function of the central nervous system in mice. MRI showed that the lateral ventricles of Pex11a^−/−^ mice were significantly larger than those of WT mice (Figure 6F and G), which is consistent with haematoxylin‐eosin staining (Figure 6H). However, there were no significant differences in the third ventricle. Furthermore, increased macrophage infiltration in the corpus callosum of Pex11a^−/−^ mice was observed compared to that of WT mice (Figure 6H).

### 3.6 Pex11a deficiency decreases peroxisome abundance in BAT

**Figure 7:**
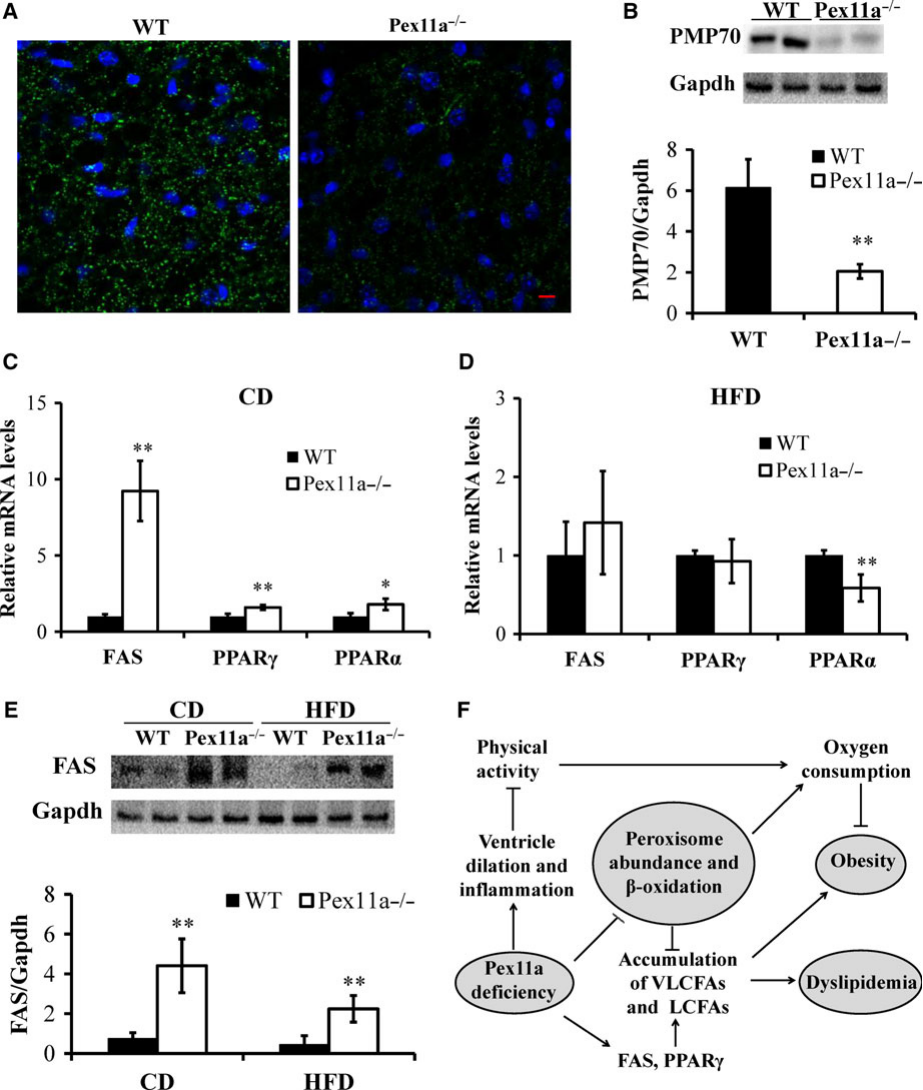
Pex11a deficiency impairs peroxisome abundance and increases de novo lipogenesis. (A) Immunofluorescence images of brown adipose tissue (BAT) of WT mice and Pex11a^−/−^ mice fed with a chow diet (CD). Peroxisomes and nuclei were stained with antibodies against peroxisome membrane protein 70 (PMP70, green) and DAPI (blue), respectively. Fluorescence emitted by Alexa Fluor 488 was visualized using a confocal laser‐scanning microscope. Scale bar: 10 μM. (B) Western blot analysis in BAT of WT mice and Pex11a^−/−^ mice fed with a CD. n = 5 per group. (C and D) Fatty acid synthase (FAS), peroxisome proliferator‐activated receptor‐α (PPARα) and PPARγ mRNAs levels in BAT of WT mice and Pex11a^−/−^ mice fed with a CD or a high‐fat diet (HFD) were measured by qRT‐PCR. n = 6 per group. (E) Western blot analysis of FAS in BAT in WT mice and Pex11a^−/−^ mice fed with a CD or an HFD for 8 weeks. n = 5 per group. Values are mean ± SD, *P < 0.05, **P < 0.01 vs WT. (F) Pex11a deficiency results in ventricular dilation and increased neroninflammation, which would decrease physical activity. Impaired physical activity needs less energy supply, which exhibits decreased energy expenditure (oxygen consumption) and results in obesity. Pex11a deficiency decreases peroxisome abundance and fatty acid β‐oxidation in adipose tissue, exhibits decreased oxygen consumption, and results in the accumulation of very long‐ and long‐chain fatty acids (VLCFAs and LCFAs); Pex11a deficiency also increases de novo lipogenesis in adipose tissue, especially FAS and PPARγ, which results in the accumulation of palmitic acid (C16:0), and then contributes to dyslipidaemia and obesity

Fatty acid β‐oxidation may be related to peroxisome abundance.^17^, ^25^ Thus, the peroxisome abundance in BAT was measured. PMP70, localized in the membrane of peroxisomes, was used to identify the peroxisomes. Immunofluorescence staining showed that the number of peroxisomes in BAT of Pex11a^−/−^ mice was lower than that in WT mice (Figure 7A). In agreement with these results, immunoblotting showed lower PMP70 protein levels in BAT of Pex11a^−/−^ mice than in BAT of WT mice (Figure 7B). These results indicate that Pex11a deficiency results in decreased peroxisome abundance, demonstrating decreased fatty acid oxidation.

### 3.7 Pex11a deficiency increases de novo fatty acid synthesis in BAT

FAS catalyses the first committed step in de novo lipogenesis, which has been implicated in obesity and insulin resistance.^26^, ^27^, ^28^ As shown in Figure 7C, on a chow diet, FAS mRNA levels in BAT of Pex11a^−/−^ mice were significantly higher than those in BAT of WT mice by approximately 10‐fold. PPARα and PPARγ mRNA expression levels of Pex11a^−/−^ mice were significantly higher than those of WT mice. Although there were no differences in FAS mRNA levels between the genotypes on an HFD, immunoblotting showed higher FAS protein levels in BAT in Pex11a^−/−^ mice on a chow diet or HFD (Figure 7E). On an HFD, PPARα mRNA levels of Pex11a^−/−^ mice were significantly lower than those of WT mice (Figure 7D). However, under the chow‐fed or starved conditions, there was no difference in hepatic FAS mRNA levels between the genotypes (Figure S1). These results indicate that Pex11a deficiency increases de novo lipogenesis in BAT but not in liver.

## 4 DISCUSSION

This study demonstrates for the first time that Pex11a deficiency is involved in ventricular dilation, which would decrease physical activity and exhibit decreased energy expenditure. Furthermore, Pex11a deficiency decreases peroxisome abundance and fatty acid β‐oxidation, and increases de novo lipogenesis in BAT, which results in accumulation of very long‐ and long‐chain fatty acids, especially in sera, and then contributes to dyslipidaemia and obesity. Our hypothesis is shown in Figure 7F.

Peroxisome biogenesis is critical for lipid metabolism and adipocyte development and function.^5^, ^21^ Previous studies showed that Pex7 deficiency tended to decrease adiposity, but Pex5 had an adverse effect, showed dysfunctional peroxisomes in adipocytes and increased fat mass.^19^, ^20^ A recent study showed that Pex16‐mediated peroxisome biogenesis in adipocytes is a target of the adipogenesis regulator‐PPARγ, and silencing Pex16 reduced peroxisome number and fatty acid oxidation, thereby causing accumulation of very long‐ and long‐chain fatty acids and triglyceride. Our results support these studies, as Pex11a^−/−^ mice have a reduced number of peroxisomes in BAT, which may be related to impaired fatty acid β‐oxidation,^17^, ^25^ accumulation of very long‐ and long‐chain fatty acids and increased fat mass. The process of fatty acid β‐oxidation in peroxisomes needs a large amount of oxygen. Therefore, decreased oxygen consumption in Pex11a ^−/−^ mice (Figure 5E‐5I) at least partially verifies impaired fatty acid β‐oxidation.

Our data also suggest that increased fat mass and dyslipidaemia may result from a marked increase in de novo lipogenesis‐related genes, especially FAS mRNA levels increased by 10‐fold. Although there are no differences in FAS mRNA levels between the genotypes under a HFD condition, immunoblotting showed that the protein levels of FAS in Pex11a^−/−^ mice were significantly higher than those in WT mice. FAS catalyses the first committed step in de novo synthesis of fatty acids that can be used for energy storage. After priming with acetyl coenzyme A, FAS uses malonyl‐CoA as a 2‐carbon source and NADPH as a cofactor to synthesize palmitate, a 16‐carbon saturated fatty acid. Our GC‐MS results demonstrate this and showed that Pex11a^−/−^ mice have significantly higher levels of palmitic acid in sera and adipose tissue. However, there are no differences in hepatic levels of FAS and palmitic acid between the genotypes, which indicate that higher levels of palmitic acid may not be derived from liver. Although the mechanism how Pex11a deficiency regulates FAS needs to be further studied, Pex11a deficiency indeed results in increased de novo lipogenesis and contributes to dyslipidaemia.

The pathology in the disorder of peroxisome biogenesis or assembly is known as peroxisome biogenesis disorders (PBDs). Zellweger spectrum is one type of PBD and, at present, has been described in patients 29 with mutations in 13 different peroxin genes (*PEX*1, *PEX2, PEX3, PEX5, PEX6, PEX10, PEX11b, PEX12, PEX13, PEX14, PEX16, PEX19, PEX26*). In patients with Zellweger spectrum, the most prominent feature is a malformation of the cortex in the brain, which results in neuronal migration defects. The phenotype of mice with Pex2, Pex5 and Pex13 deficiencies resembles the features of human Zellweger syndrome, showing severe hypotonia and cerebral and cerebellar malformation.^30^, ^31^, ^32^ Pex11b deficiency in mice results in several pathological features shared by Zellweger syndrome, including neuronal migration defects, enhanced neuronal apoptosis, developmental delay, hypotonia and neonatal lethality.^33^ In the present study, another Pex11 family member, Pex11a, which acts as a membrane elongation factor during peroxisome proliferation,^17^, ^25^ may be involved in brain pathology. Its deficiency results in ventricular dilation and increased macrophage infiltration, indicating malformation of the cerebrum (Figure 6), which may be because of decreased peroxisome abundance in cerebrum (data not shown). However, whether Pex11a deficiency causes accumulation of very long‐chain fatty acids in the brain and then results in ventricular dilation and macrophage infiltration, needs further studied.

Decreased physical activity and motor function was observed in Pex11a^−/−^ mice, especially remaining quiescent during the shift from night to day, compared to those in WT mice. This observation may be at least partially explained by the ventricular dilation and increased macrophage infiltration in Pex11a^−/−^ mice, which is similar to the disorder of peroxisome biogenesis caused by other peroxin genes. Mice with knockout of Pex5 in oligodendrocytes exhibited axonal degeneration, progressive subcortical demyelination and neuroninflammation, and gradually developed impaired motor function.^34^ Our results were supported by another study, which showed that mice exhibit ventricular dilation and severe hydrocephalus, resulting in decreased motor function and motor learning ability.^35^ Decreased physical activity may be related to decreased system energy expenditure in Pex11a^−/−^ mice (Figures 5 and 6), which were also consistent with decreased levels of fatty acid oxidation‐relative gene (PPARα), resulting in accumulation of very long‐ or long‐chain fatty acids. This finding may explain for the lipid metabolism dysregulation and obesity in Pex11a^−/−^ mice. However, Li et al^23^ reported that mice lacking Pex11a developed normally, showing normal levels of very long‐chain fatty acids in plasma. The reason for this difference is presently unclear. However, in the present study, HFD and older mice (20 weeks old) were investigated. Pex11a is an inducible gene, and phenotypic differences in WT mice and Pex11a^−/−^ mice will be more evident under HFD and older conditions. This may account for the differences between the study of Li et al^23^ and our results. Furthermore, although there were no differences in blood glucose levels and glucose tolerance between WT and Pex11a^−/−^ mice on a chow diet,^17^ in the present study, feeding an HFD elicited a phenotypic difference.

In summary, the current study suggests that Pex11a is required for peroxisome proliferation in BAT and highlights the relation between Pex11a and dyslipidaemia and obesity. Understanding of the mechanisms by which Pex11a deficiency decreased energy expenditure and fatty acid oxidation and resulted in the accumulation of very long‐ and long‐chain fatty acids will be required to develop therapeutic strategies for dyslipidaemia and obesity.

## CONFLICTS OF INTEREST

The authors confirm that there is no conflict of interests.
