# Corrigendum

**DOI:** 10.1111/jcmm.16042

**Published:** 2020-12-08

In the article,^1^ the affiliations of the two authors were incorrect.

The affiliation of Yi Tan should be ^6^Pediatric Research Institute, Departments of Pediatrics, Radiation Oncology, Pharmacology and Toxicology, University of Louisville, Louisville, Kentucky.

The affiliation of Lu Cai should be ^6^Pediatric Research Institute, Departments of Pediatrics, Radiation Oncology, Pharmacology and Toxicology, University of Louisville, Louisville, Kentucky.

Both authors are not affiliated to

^1^Chinese‐American Research Institute for Pediatrics & Department of Pediatrics, The First Affiliated Hospital of Wenzhou Medical University, Chashan University‐ Town, Wenzhou, China.

^4^Department of Pharmaceutical Sciences, Wenzhou Medical University, Wenzhou, China.
