# Supplementary material for: Pex11a deficiency causes dyslipidaemia and obesity in mice
Source: J Cell Mol Med. 2018 Dec 25;23(3):2020–31. doi: 10.1111/jcmm.14108 (PMC6378206; doi:10.1111/jcmm.14108)
Supplement: Supplementary file 1 [file JCMM-23-2020-s001.pdf]

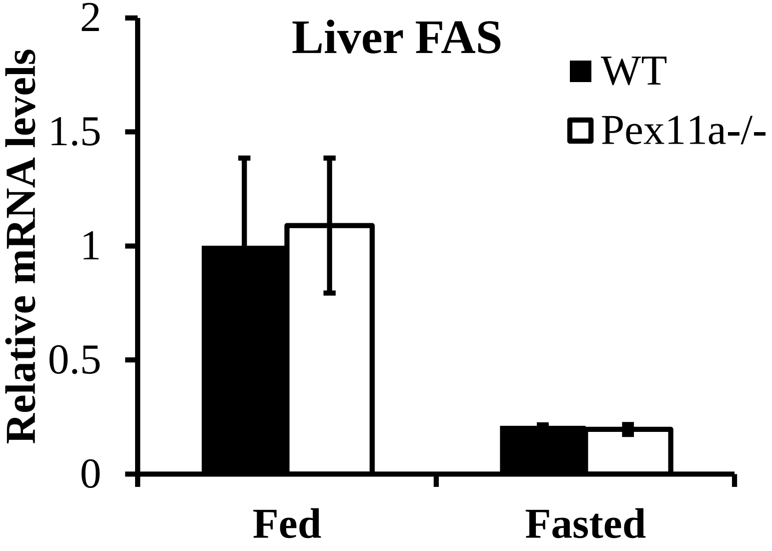


Figure S1. The relative fatty acid synthase (FAS) mRNAs levels in the livers of WT mice and Pex11a$^{-/-}$ mice fed with a chow diet or fasted for 24 hours were measured by qRT-PCR.
