# Supplementary material for: Pex11a deficiency causes dyslipidaemia and obesity in mice
Source: J Cell Mol Med. 2018 Dec 25;23(3):2020–31. doi: 10.1111/jcmm.14108 (PMC6378206; doi:10.1111/jcmm.14108)
Supplement: Supplementary file 2 [file JCMM-23-2020-s002.docx]

Table S1 The sequences of oligonucleotide primers of mouse genes for RT-PCR

| Gene | Forward Primer 5’ to 3’ | Reverse Primer 5’ to 3’ |
| --- | --- | --- |
| FAS | GGAGGTGGTGATAGCCGGTAT | TGGGTAATCCATAGAGCCCAG |
| PPARα | CCAGATGTGCCTGCTGCTTCC | TGGTCGGTCTACAGAGTGAGTTCC |
| PPARγ | ATGGCGACAAGCACAGACTACAAC | GAGCATCAAGGCAGCGGACTTAC |
| GAPDH | CCACTTTTGATGCTGGGGCT | TGAGCTTGACGAAGTGGTCG |
